# Barrier and no-touch techniques in gastrointestinal surgery: a best evidence summary

**DOI:** 10.3389/fsurg.2026.1948728

**Published:** 2026-10-12

**Authors:** Qingqing Du, Hailei Bian, Shan Wu, Fangyuan Sun, Li Ni

**Affiliations:** 1 Tongji University School of Medicine, Shanghai, China; 2 Shanghai Children’s Hospital, School of Medicine, Shanghai Jiao Tong University, Shanghai, China; 3 Shanghai East Hospital, School of Medicine, Tongji University, Shanghai, China

**Keywords:** barrier technique, gastrointestinal surgery, infection prevention, no-touch isolation technique, perioperative management, surgical site infection

## Abstract

**Objective:**

To systematically summarize the best available evidence on intraoperative barrier and no-touch techniques for the prevention of surgical site infection (SSI) in adult patients undergoing gastrointestinal (GI) surgery, and to provide an evidence base for developing standardized clinical practice protocols.

**Methods:**

Guided by the “6S” evidence model, a systematic literature search was conducted across multiple databases from their inception to March 1, 2026. The search encompassed all evidence pertaining to intraoperative barrier and no-touch techniques in GI surgery, including guideline organization websites (WHO, CDC, NICE, SHEA/IDSA, APIC, GIN, NGC, SIGN, RNAO, and medlive.cn), evidence-based databases (The Cochrane Library and JBI Evidence Synthesis), the trial registry ClinicalTrials.gov, and the databases PubMed, Web of Science, Embase, CINAHL, CBM, CNKI, VIP, and WanFang. Included studies comprised guidelines, expert consensus statements, evidence summaries, systematic reviews, narrative reviews, and randomized controlled trials (RCTs). Quality assessment, data extraction, and evidence grading were independently performed by two researchers trained in evidence-based nursing.

**Results:**

A total of 18 publications were included: 5 guidelines, 3 expert consensus statements, 1 evidence summary, 3 systematic reviews, 3 narrative reviews, and 3 RCTs. Thirty best-evidence statements were synthesized across six domains: no-touch isolation technique (NTIT)–derived contamination control, intraoperative aseptic barrier precautions, intraoperative wound protection, selection of intraoperative irrigation solutions, intraoperative temperature management, and surgical teamwork and instrument management.

**Conclusions:**

This evidence summary provides a standardized, SSI-focused framework for barrier and no-touch practices in GI surgery. High-level evidence supports wound protectors, mechanical bowel preparation plus oral antibiotics in elective colorectal surgery, and intraoperative normothermia, whereas most NTIT-derived contamination-control practices are supported mainly by expert-consensus evidence and are recommended conditionally (Grade B). Clinical translation should be guided by the FAME (Feasibility, Appropriateness, Meaningfulness, and Effectiveness) framework and tailored to local hospital conditions and individual patient characteristics to minimize the risk of SSI.

## 1 Introduction

Surgical site infection (SSI) is one of the most common healthcare-associated infections after gastrointestinal (GI) surgery, accounting for approximately 15%–20% of all such infections (1). Because GI surgery involves entry into the digestive tract, most surgical wounds are classified as clean-contaminated (Class II) or contaminated (Class III), and reported SSI rates after colorectal surgery range from 5% to 26%—far higher than after clean surgery (2). In surgery for GI malignancies, intraoperative tumor cell shedding additionally motivates the no-touch isolation technique (NTIT), although high-quality trials have not confirmed an oncologic survival benefit over conventional techniques (3).

For terminology consistency, “barrier techniques” in this manuscript denote the bundle of intraoperative measures that isolate the surgical field from microbial contamination (hand hygiene, standard precautions, aseptic practice, wound protection, and environmental management), whereas “no-touch isolation technique (NTIT)” refers specifically to measures that avoid direct contact with tumor and contaminated tissue; these two terms are used consistently throughout. Clinical nurses vary considerably in their knowledge and implementation of these techniques, and standardized operational guidance for GI surgery remains lacking. This best evidence summary therefore used an evidence-based nursing methodology to systematically identify, critically appraise, and synthesize the best available evidence on barrier and no-touch techniques for SSI prevention in GI surgery, thereby providing an evidence base for clinical nursing practice.

Although barrier techniques and the NTIT arose from different historical rationales—microbial contamination control and SSI prevention for the former, and minimization of direct tumor manipulation and potential tumor-cell dissemination for the latter (3)—we integrated them into a single research question and evidence framework for three reasons. First, the two approaches converge mechanistically and operationally in GI surgery: the critical moments of microbial contamination (opening of the bowel lumen, handling and retrieval of specimens, anastomosis and reconstruction, and incision closure) are precisely the moments at which NTIT-derived practices (zoned “clean–contaminated” instrument management, glove and instrument changes, and specimen isolation) (4–6) are applied, and both sets of practices are executed by the same surgical and nursing team as one continuous intraoperative workflow. Second, because high-quality evidence has not confirmed an oncologic survival benefit of the NTIT (3, 7), the principal contemporary justification for retaining NTIT-derived practices is their contribution to contamination control and SSI prevention; evaluating them under a common SSI-focused outcome therefore aligns the research question with the outcome for which the supporting evidence is strongest. Third, from a clinical nursing perspective, operating room nurses require a single, coherent operational protocol rather than two conceptually separate rule sets; integrating these conceptually distinct but operationally overlapping interventions into one SSI-focused framework reflects how they are actually implemented in practice and avoids fragmented and inconsistent application.

Regarding the novelty of this evidence synthesis, we acknowledge that individual barrier-related interventions—such as aseptic technique, wound protection, and intraoperative temperature management—have been extensively evaluated and incorporated into established SSI prevention guidelines (e.g., WHO (8), CDC (9), SHEA/IDSA (10), NICE (11), and ASCRS (4). However, important evidence gaps remain unresolved by these guidelines: they address SSI prevention for surgical patients in general or for selected procedures, provide limited GI-specific and minimally invasive or robotic surgery-specific operational detail, are dispersed across multiple documents that clinicians must consult separately, and are written primarily from a surgical rather than a nursing implementation perspective, offering little graded, step-by-step guidance on how barrier and no-touch practices should be operationalized, sequenced, and quality-controlled throughout the entire intraoperative process; moreover, existing SSI guidelines do not systematically address NTIT-derived practices, whose role warrants reappraisal specifically against SSI outcomes (3, 7). The additional clinical and scientific value of this Best Evidence Summary beyond currently available SSI prevention recommendations is therefore threefold: (1) it consolidates scattered recommendations into 30 graded, GI-specific best-evidence statements covering the whole intraoperative workflow, including NTIT-derived contamination-control practices; (2) it critically reappraises and grades each statement using JBI levels of evidence and FAME-based recommendation strength, distinguishing high-certainty “core” measures from lower-certainty “flexible” ones; and (3) it translates the evidence into actionable, tiered implementation strategies for operating room nurses across different hospital levels, thereby providing an operational evidence base for standardized GI-surgery nursing protocols that existing guidelines do not offer.

## 2 Methods

### 2.1 Question formulation

The PIPOST framework was applied to formulate the evidence-based question as follows: Population (P) included adult patients (≥18 years) undergoing elective or emergency GI surgery—open or minimally invasive procedures such as radical gastrectomy, radical colorectal resection, and intestinal obstruction surgery—for benign or malignant disease; Intervention (I) involved surgical barrier techniques (e.g., hand hygiene, standard precautions, aseptic technique, NTIT-derived contamination-control practices, wound protection, intraoperative irrigation, and intraoperative temperature management); Professionals (P) comprised healthcare personnel participating in GI surgery; Outcomes (O) included the incidence of SSI and pass rates for theoretical knowledge and practical application of barrier and no-touch techniques among operating room nurses; Setting (S) encompassed operating rooms at various hospital levels; and Type of evidence (T) consisted of guidelines, evidence summaries, systematic reviews, expert consensus statements, narrative reviews, and randomized controlled trials.

### 2.2 Search strategy

#### 2.2.1 Information sources

Following the “6S” evidence pyramid model, a top-down literature search was conducted across the following resources: international organizations and guideline websites (WHO, CDC, NICE, SHEA/IDSA, APIC, GIN, NGC, SIGN, RNAO, and medlive.cn); evidence-based databases (The Cochrane Library and JBI Evidence Synthesis); trial registries (ClinicalTrials.gov); English-language databases (PubMed, Web of Science, Embase, and CINAHL); and Chinese databases (CBM, CNKI, VIP, and WanFang).

#### 2.2.2 Search terms

The search terms included: gastrointestinal surgery/digestive tract surgery/abdominal surgery/colorectal surgery; surgical barrier/barrier technique/aseptic technique/no-touch isolation technique/infection control/intraoperative infection prevention/surgical site infection prevention/wound protector; and perioperative nursing/intraoperative care/surgical nursing/operating room nursing.

The search period extended from database inception to March 1, 2026.

### 2.3 Inclusion and exclusion criteria

#### 2.3.1 Inclusion criteria

The inclusion criteria were as follows: (1) adult patients (≥18 years) undergoing elective or emergency, open or minimally invasive GI surgery involving the stomach, small intestine, colorectum, and other GI organs, for benign or malignant indications; (2) studies evaluating at least one intraoperative barrier or protective measure implemented in the operating room to prevent contamination and SSI; (3) clinical decision support documents, guidelines, expert consensus statements, evidence summaries, systematic reviews, meta-analyses, narrative reviews, and randomized controlled trials (RCTs); (4) publications in Chinese or English; and (5) when multiple versions of similar evidence were available, studies published between 2021 and 2026 were preferentially included.

#### 2.3.2 Exclusion criteria

The exclusion criteria were as follows: (1) publications written in languages other than Chinese or English; (2) studies for which the full text was unavailable; and (3) studies that did not meet the predefined quality appraisal criteria. All retrieved records were independently screened by two researchers trained in evidence-based methodology. Any disagreements were resolved through discussion until consensus was reached; if consensus could not be reached, a third researcher trained in evidence-based methodology was consulted for arbitration.

### 2.4 Quality appraisal tools

Guidelines were assessed using the Appraisal of Guidelines for Research and Evaluation II (AGREE II) instrument. Expert consensus statements and narrative reviews were appraised using the 2016 Joanna Briggs Institute (JBI) Critical Appraisal Checklist for Text and Opinion Papers. Systematic reviews and RCTs were evaluated using the corresponding 2016 JBI critical appraisal checklists. Study designs were classified according to the design reported by the original authors and verified against the full text; evidence syntheses without a systematic search and critical appraisal of primary studies were classified as narrative reviews rather than systematic reviews.

### 2.5 Evidence extraction, synthesis, and grading

Two researchers trained in evidence-based nursing independently performed literature screening, quality appraisal, evidence extraction, and evidence grading. Evidence was initially graded using the 2014 JBI Levels of Evidence and Grades of Recommendation for Intervention Studies. To make the grading process reproducible, the following rules were prespecified before extraction: (1) each evidence statement was assigned the level of its highest-level supporting source; (2) statements supported directly by a systematic review or meta-analysis of RCTs were graded 1a, and those supported by an individual RCT were graded 1b; (3) statements derived from evidence-based guidelines were assigned the evidence level reported for the corresponding guideline recommendation, and where the guideline did not report an evidence level—or where a statement was supported only by expert consensus statements or narrative reviews—the statement was graded 5b (expert consensus); (4) recommendation strength was then determined independently by the two researchers according to the FAME (Feasibility, Appropriateness, Meaningfulness, and Effectiveness) framework, with Grade A (strong recommendation) assigned only when all four FAME criteria were judged to be satisfied and Grade B (weak recommendation) assigned otherwise; and (5) any disagreement in grading was resolved by a third evidence-based nursing expert. When discrepancies existed among different sources of evidence, priority was given to evidence from higher levels of the evidence hierarchy, studies of superior methodological quality, and the most recent authoritative publications.

## 3 Results

### 3.1 Literature search results

A total of 18 publications were ultimately included, comprising 5 guidelines, 3 expert consensus statements, 1 evidence summary, 3 systematic reviews, 3 narrative reviews, and 3 RCTs. The literature screening process is illustrated in Figure 1, and the characteristics of the included studies are summarized in Table 1.

**Figure 1:**
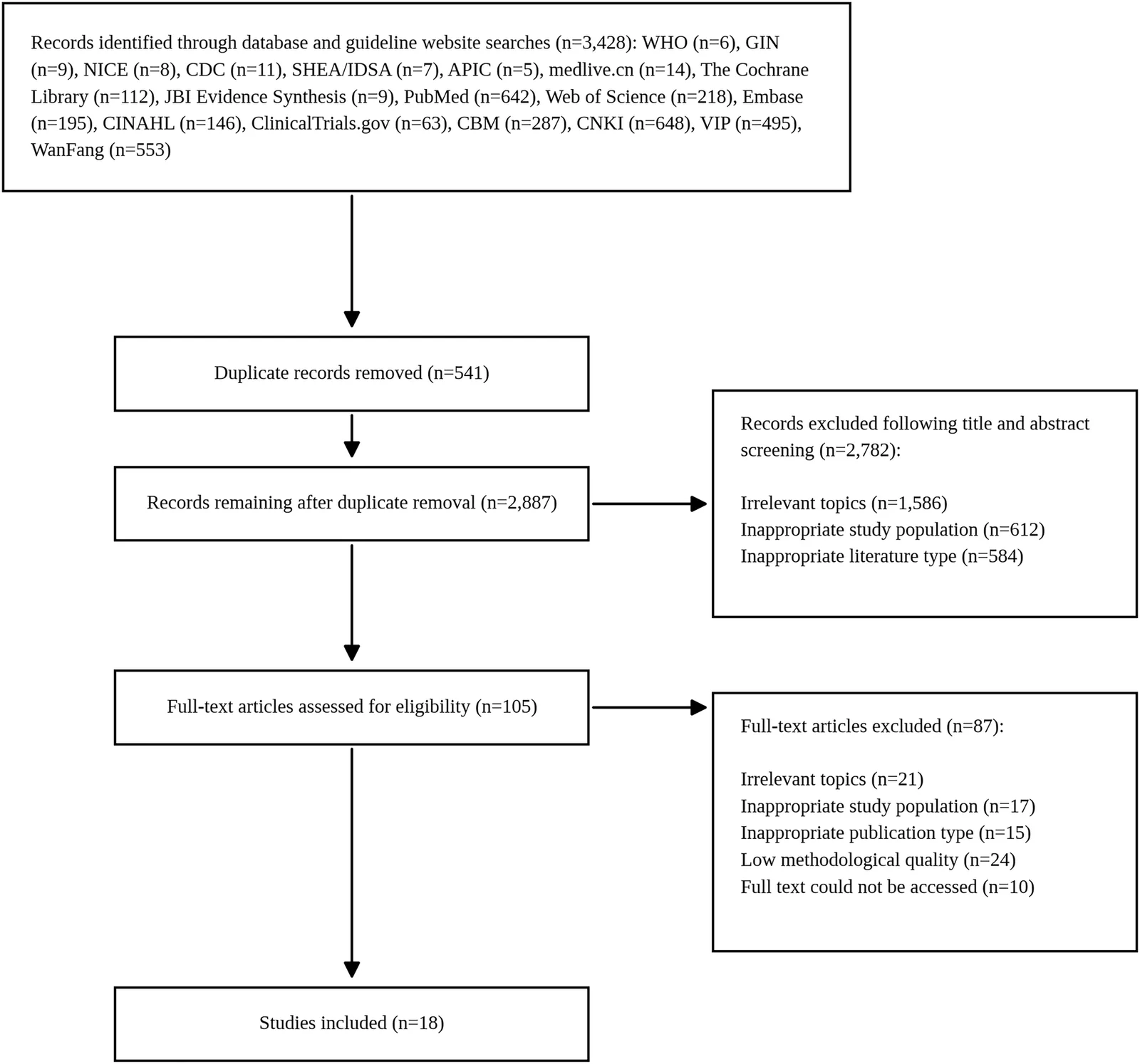
Literature screening flow diagram.

**Table 1:** Characteristics of the included studies (*n* = 18).

ID	Title	Year	Country/Organization	Topic	Source/Publisher	Evidence Type	Patient population and surgical procedures	Clinical setting and primary outcomes
G1 (10)	Strategies to prevent SSI in acute-care hospitals: 2022 Update	2023	USA	SSI prevention strategies	SHEA/IDSA/APIC	Guideline	Surgical inpatients; all procedures	Acute-care hospitals, USA; SSI incidence
G2 (11)	SSI: Prevention and Treatment (NG125 Update)	2024	UK	SSI prevention and treatment	NICE	Guideline	Surgical patients (all ages); all specialties	UK hospital surgical settings; SSI prevention and treatment
G3 (8)	Global guidelines for the prevention of SSI (2nd ed.)	2018	WHO	Global SSI prevention guidelines	WHO	Guideline	Surgical patients worldwide; all procedures	Global, all resource settings; SSI incidence
G4 (4)	Preventing SSI (2024)	2024	USA	SSI prevention in colorectal surgery	ASCRS	Guideline	Adult colorectal surgery patients; colorectal resection	Hospitals, USA; SSI in colorectal surgery
G5 (21)	Strategies to prevent healthcare-associated infections through hand hygiene: 2022 Update	2023	USA	Hand hygiene for healthcare-associated infection prevention	SHEA/IDSA/APIC	Guideline	Acute-care hospital patients; hand hygiene practice	Acute-care hospitals, USA; HAI incidence
C1 (12)	Expert consensus on prevention and management of perioperative hypothermia (2023 edition)	2023	China	Perioperative hypothermia prevention and management	Medical Journal of Peking Union Medical College Hospital	Expert consensus	Perioperative adults; all procedures	Operating rooms, China; perioperative hypothermia and complications (incl. SSI)
C2 (13)	Chinese clinical practice guidelines for enhanced recovery after surgery (2021 edition)	2021	China	ERAS and perioperative management	Chinese Journal of Practical Surgery	Expert consensus	Adults undergoing abdominal/GI surgery; elective GI procedures	Hospitals, China; recovery and complications (incl. SSI)
C3 (5)	Guidelines in practice: sterile technique	2024	USA	Sterile technique and surgical barrier practice	AORN Journal	Expert consensus	Surgical patients; all operative procedures	Operating rooms, USA (AORN); adherence to sterile technique and SSI prevention
E1 (14)	Evidence analysis of SSI prevention in adult inpatients based on guidelines and clinical decisions	2024	China	Evidence summary of SSI prevention in adult patients	Chinese Evidence-Based Nursing	Evidence summary	Adult inpatients; all surgical procedures	Hospitals, China; SSI incidence
SR1 (15)	Perioperative care bundles for the prevention of surgical-site infections: meta-analysis	2022	Netherlands	Perioperative bundles for SSI prevention	British Journal of Surgery	Systematic review	Surgical patients across specialties; various procedures	Hospitals; SSI after care bundles
SR2 (16)	Incisional wound irrigation for the prevention of SSI: a systematic review and network meta-analysis	2024	Netherlands	Wound irrigation for SSI prevention	JAMA Surgery	Systematic review	Patients with incisional wounds; laparotomy and other procedures	Hospitals; SSI
SR3 (17)	Role of wound protectors in preventing SSI in patients undergoing abdominal surgery: a meta-analysis of RCTs	2025	China	Wound protectors for SSI prevention	BMC Surgery	Systematic review	Abdominal surgery patients (19 RCTs, 4,779); abdominal/GI procedures	Hospitals; SSI
NR1 (6)	Essential updates 2022–2023: surgical and adjuvant therapies for locally advanced colorectal cancer	2024	Japan	Colorectal cancer surgery and adjuvant therapy updates (including NTIT review)	Annals of Gastroenterological Surgery	Narrative review	Locally advanced colorectal cancer patients; colorectal resection (incl. NTIT)	Hospitals, Japan; oncologic outcomes and complications
NR2 (18)	SSI prevention: a review	2023	USA	Comprehensive review of SSI prevention	JAMA	Narrative review	Surgical patients; all procedures	Hospitals, USA; SSI and prevention outcomes
NR3 (22)	SSI prevention: best practices and new approaches	2025	Saudi Arabia	Best practices and new approaches for SSI prevention	International Journal of Community Medicine and Public Health	Narrative review	Surgical patients; all surgical procedures	Hospitals, Saudi Arabia; SSI and preventive practices
R1 (19)	Plastic wound protector vs surgical gauze for SSI reduction in open GI surgery: a randomized clinical trial	2024	Republic of Korea	Wound protector vs gauze in open GI surgery	JAMA Surgery	RCT	Adults (18–75 y) undergoing elective or emergent open GI surgery (*n* = 458); stomach, small intestine, colon and rectum	Multicenter (13 hospitals), Republic of Korea; 30-day SSI (protector vs gauze)
R2 (20)	Intraoperative wound irrigation for the prevention of SSI after laparotomy: a randomized clinical trial by CHIR-Net	2024	Germany	RCT of intraoperative wound irrigation (polyhexanide vs saline vs no irrigation)	JAMA Surgery	RCT	Adults undergoing laparotomy (*n* = 689)	Multicenter, Germany (CHIR-Net); 30-day SSI (polyhexanide vs saline vs no irrigation)
R3 (7)	Long-term follow-up of the conventional versus NTIT for resection of primary colon cancer (JCOG1006): randomized clinical trial	2024	Japan	Long-term follow-up of NTIT	BJS Open	RCT	Primary colon cancer patients (*n* = 853); resection (NTIT vs conventional)	Multicenter, Japan; disease-free and overall survival

### 3.2 Quality appraisal results

The quality appraisal results are presented in Tables 2–5.

**Table 2:** Quality appraisal of guidelines (*n* = 5).

Guideline	Scope & purpose (%)	Stakeholder involvement (%)	Rigor of development (%)	Number of domains ≥60%	Number of domains ≥30%
G1 (10)	92	78	85	6	6
G2 (11)	89	72	82	6	6
G3 (8)	94	83	90	6	6
G4 (4)	88	75	80	6	6
G5 (21)	88	74	71	6	6

**Table 3:** Quality appraisal of expert consensus statements and narrative reviews (*n* = 6).

Consensus	Q1	Q2	Q3	Q4	Q5	Q6
C1 (12)	Yes	No	Yes	Yes	Yes	Yes
C2 (13)	Yes	Yes	Yes	Yes	Yes	No
C3 (5)	Yes	Yes	Yes	Yes	Yes	No
NR1 (6)	Yes	Yes	Yes	Yes	Yes	Yes
NR2 (18)	Yes	Yes	Yes	Yes	Yes	Yes
NR3 (22)	Yes	No	No	Yes	Yes	No

Q1: Clear source of opinions; Q2: Opinions based on analysis; Q3: Derived from influential experts; Q4: Centered on the interests of relevant populations; Q5: Logically expressed opinions; Q6: Consistent with previous literature.

**Table 4:** Quality appraisal of systematic reviews (*n* = 3).

Review	Q1	Q2	Q3	Q4	Q5	Q6	Q7	Q8	Q9	Q10	Q11
SR1 (15)	Yes	Yes	Yes	Yes	Yes	Yes	Yes	Yes	Yes	Yes	Yes
SR2 (16)	Yes	Yes	Yes	Yes	Yes	Yes	Yes	Yes	Yes	Yes	Yes
SR3 (17)	Yes	Yes	Yes	Yes	Yes	Yes	Yes	Yes	Yes	Yes	Yes

Q1: Clear research question; Q2: Appropriate inclusion criteria; Q3: Appropriate search strategy; Q4: Sufficient information sources; Q5: Appropriate quality appraisal criteria; Q6: Appraisal by ≥2 reviewers; Q7: Appropriate synthesis methods; Q8: Publication bias assessed; Q9: Conclusions supported by analysis; Q10: Heterogeneity fully discussed; Q11: Appropriate recommendations.

**Table 5:** Quality appraisal of RCTs (*n* = 3).

RCT	Q1	Q2	Q3	Q4	Q5	Q6	Q7	Q8	Q9	Q10	Q11	Q12	Q13
R1 (19)	Yes	Yes	Yes	Yes	Unclear	Yes	Yes	Yes	Yes	Yes	Yes	Yes	Yes
R2 (20)	Yes	Yes	Yes	Yes	Yes	Yes	Yes	Yes	Yes	Yes	Yes	Yes	Yes
R3 (7)	Yes	Yes	Yes	No	No	Unclear	Yes	Yes	Yes	Yes	Yes	Yes	Yes

Q1: True randomization; Q2: Allocation concealment; Q3: Comparable baseline between groups; Q4: Blinding of participants; Q5: Blinding of intervention providers; Q6: Blinding of outcome assessors; Q7: Identical interventions across groups; Q8: Complete follow-up; Q9: Intention-to-treat analysis; Q10: Identical outcome measurement methods across groups; Q11: Reliable outcome measurement methods; Q12: Appropriate data analysis methods; Q13: Appropriate study design.

#### 3.2.1 Guidelines

The AGREE II assessment revealed standardized scores of ≥60% in all domains for the five guidelines, indicating high overall quality. All guidelines were graded A and recommended for direct clinical application.

#### 3.2.2 Expert consensus statements and narrative reviews

The JBI appraisal indicated that consensus statement C112 fulfilled all six criteria. C213 and C35 did not fulfill criterion Q6 (consistency with previous literature). C2 represents a comprehensive ERAS guideline, of which only some sections are directly related to barrier techniques. C3 addresses general sterile techniques for operating rooms without specific reference to GI surgical barriers; yet, its core recommendations concerning hand hygiene, aseptic technique, and glove changes align well with specialty guidelines. Among the narrative reviews, NR16 and NR218 fulfilled all six criteria, whereas NR322 did not fulfill criteria Q2 (opinions based on analysis) and Q6 (consistency with previous literature); evidence from NR3 was therefore used only to corroborate, never to solely support, an evidence statement. Overall, the quality of all three consensus statements was acceptable, and thus all were included.

#### 3.2.3 Evidence summary

The single included evidence summary thoroughly traced and evaluated its original references, demonstrating high overall quality, and was therefore included.

#### 3.2.4 Systematic reviews

#### 3.2.5 RCTs

### 3.3 Evidence synthesis, grading, and recommendations

Content analysis of the included studies was conducted with a focus on SSI prevention and intraoperative contamination control. Thirty best-evidence statements were synthesized across six domains. Evidence grades and recommendation strengths are presented in Table 6. After grading under the prespecified rules, three statements—wound protection, mechanical bowel preparation plus oral antibiotics for elective colorectal surgery, and intraoperative temperature management—were supported by level-1 evidence and assigned Grade A recommendations. Most NTIT-derived contamination-control statements were supported by expert-consensus evidence (level 5b) and were accordingly downgraded to Grade B, reflecting the absence of direct RCT evidence for these practices against SSI outcomes.

**Table 6:** Summary of best-evidence statements.

Domain	No	Evidence statement	Evidence level	Recommendation strength
1. NTIT-Derived Contamination Control	1	Surgical teams should reinforce awareness of SSI prevention and intraoperative contamination control, establish a multidisciplinary collaboration mechanism, and strengthen preoperative communication and information sharing among medical and nursing teams (6, 7, 17).	5b	B
	2	A clear barrier-designated zone should be established within the sterile field, and the surgical instrument table should be divided into a “contaminated zone” and a “clean zone”. Barrier measures should be initiated once contaminated or tumor-related manipulation of the digestive tract is performed (5, 6).	5b	B
	3	After barrier procedures, the sterile field should be reset. Gloved sterile hands should not directly contact contaminated sources; contaminated instruments and dressings should be placed in the designated contaminated zone and should not be reused on normal tissues (4, 5).	5b	B
	4	Gloves should be changed after abdominal exploration in open surgery. After tumor resection and before digestive tract reconstruction, all surgical personnel should change outer gloves and use new surgical instruments (4–6).	5b	B
	5	Gloves should be changed before abdominal incision closure/before abdominal fascial layer closure. For combined abdominoperineal surgery, perineal operators participating in abdominal procedures should re-perform hand disinfection and change gloves and surgical gowns (4, 5).	5b	B
	6	Intraoperatively resected specimens should be immediately placed in dedicated specimen bags or containers; direct hand contact with tumor tissue should be avoided. Specimen retrieval bags are single-use only. For natural orifice specimen extraction surgery (NOSES), adequate irrigation and mechanical protection of the extraction tract should be performed in accordance with institutional protocols and device instructions; the previous fixed mandate for repeated iodophor irrigation was removed because it is not supported by contemporary SSI-prevention evidence (5, 6, 23).	5b	B
	7	Resected surgical specimens should be covered and moved to a separate area away from the instrument table. Postoperative specimen handling should not be performed within the sterile field. Instruments, blades and dressings used for sampling multiple lesions to be tested should be changed for each lesion site (5, 6).	5b	B
2. Intraoperative Aseptic Barrier Precautions	8	Medical and nursing personnel participating in GI surgery should receive standardized training on surgical barrier techniques, and may only participate in surgical cooperation after passing theoretical and practical assessments. Operating rooms should establish quality control teams for surgical barrier measures to regularly assess the work quality of nursing staff (4, 5).	5b	A
	9	Preoperative patient baseline conditions should be assessed. For elective colorectal surgery, mechanical bowel preparation combined with oral antibiotics (MBP + OAB) should be completed in accordance with guideline recommendations, and the adequacy of bowel preparation should be evaluated before the procedure; the Boston Bowel Preparation Scale cited in the previous version was removed because it is a colonoscopy-specific tool rather than an intraoperative assessment instrument (4, 8, 22).	1a	A
	10	During preoperative visits, ascites volume may be assessed based on imaging data. Targeted barrier plans may be developed for patients with large-volume ascites (4, 6).	5b	B
	11	Standard precautions should be implemented for all patients undergoing GI surgery. When operations may cause fluid splashing, additional face shields/goggles and fluid-resistant barrier gowns should be worn (5, 8, 10).	5b	A
	12	The surgical team should wear double gloves throughout the procedure; gloves should be changed immediately if perforated. Gauze balls used for skin disinfection are single-use only (4).	5b	A
	13	Gauze dressings that have contacted GI contents or tumor tissue are single-use only; wet gauze used for wiping instruments should be used separately; used gauze in laparoscopic surgery should be placed in specimen retrieval bags (5).	5b	A
	14	Instruments that have contacted GI contents should not be used on clean surgical tissues; perineal operation instruments should be used exclusively; instruments for intraoperative lymph node dissection should be dedicated; instruments for specimen retrieval should be used separately (5).	5b	A
3. Intraoperative Wound Protection	15	After laparotomy, an appropriate wound protector (double-ring plastic type preferred) should be selected according to incision size and applied to protect the wound edges, and additional sterile drapes should be placed under the incision to reduce the risk of SSI. Routine use of plastic adhesive incise drapes is no longer recommended for SSI prevention, consistent with WHO and NICE guidance (4, 8, 11, 17, 19).	1a	A
	16	For laparoscopic surgery, disposable non-slip trocars should be provided, or trocars should be fixed to the abdominal wall; the use of specimen retrieval bags is strongly recommended when retrieving specimens through trocar sites to avoid direct contact between tumor tissue and the incision (5, 6, 23).	5b	A
4. Intraoperative Irrigation Fluid Selection	17	The benefit of routine peritoneal or incisional irrigation for SSI prevention is uncertain: a multicenter RCT and a network meta-analysis found no significant SSI reduction with saline or antiseptic irrigation compared with no irrigation. When irrigation is clinically indicated (e.g., to remove gross contamination), warm (37℃–40℃) sterile normal saline should be used; irrigation with sterile water for injection solely for SSI prevention is no longer recommended (9, 16, 20).	1b	B
	18	For transanal anastomosis, a mucosal disinfectant solution should be provided for tract irrigation; after peritoneal suture is completed, the incision and trocar removal sites should be irrigated with sterile normal saline (12, 16).	5b	B
	19	In case of tumor rupture or GI content spillage: (1) immediately aspirate spilled contents or exfoliated tissues with a suction device; (2) cover the rupture margins or contaminated areas with disinfectant-soaked gauze dressings; and (3) irrigate the contaminated area with 37℃–40℃ sterile normal saline (4, 8).	5b	A
5. Intraoperative Temperature Management	20	For GI surgery, sufficient warming preparation should be completed before abdominal opening. Intra-abdominal exposure time should be minimized as much as possible; exposed bowel should be covered with warm moist gauze, and core body temperature should be maintained at ≥36.0℃ (4, 12, 13).	1a	A
6. Surgical Cooperation and Instrument Management	21	Operating instruments and equipment should be prepared according to surgical approaches. Replaceable surgical instruments and disposable items should be equipped according to the surgical procedure, and an additional sterile instrument cart may be prepared for perineal procedures (4, 5).	5b	A
	22	Two suction devices are recommended for GI surgery, used for suctioning GI contents and peritoneal irrigation fluid respectively, to avoid cross-contamination (8, 10).	5b	B
	23	Physical barrier preparation should include: gauze dressings, wound protection devices (wound protectors/retrieval bags/medical sterile protective sheaths) prepared according to surgical approaches; ostomy care supplies should be fully prepared for enterostomy surgery (4, 5, 17).	5b	B
	24	Additional sterile surgical drapes should be placed around the incision, and fluid-resistant surgical drapes are preferred. During digestive tract reconstruction and before incision closure, additional sterile surgical drapes should be placed to re-establish the sterile area (4).	5b	B
	25	Before incising GI tissue, gauze dressings should be provided to protect surrounding normal tissues; the anastomotic ends of GI tissue should be wrapped with gauze dressings; resected surgical specimens should be wrapped with gauze dressings, placed in a specimen basin and moved to the designated contaminated area (5).	5b	A
	26	Before opening the GI lumen, bowel clamps or closure devices should be used to seal both ends of the lumen. For NOSES, ties or closure devices should be provided to seal both ends of the intestinal lumen. A suction device and disinfectant-soaked gauze dressings should be prepared before incision (4, 5, 17).	5b	A
	27	Surgical instruments that have contacted GI contents or tumor tissue should be removed from the sterile field and must not be reused on clean tissue after on-field rinsing or soaking in antiseptic solutions; they should be collected separately and undergo standardized cleaning, disinfection, and sterilization before any reuse. Stapler cartridges should be wrapped in gauze during disassembly, and used staplers should be discarded under the surgical table (4, 5).	5b	A
	28	For laparoscopic surgery, pneumoperitoneum pressure should be set within the standard laparoscopic range, preferably ≤14 mmHg with a flow rate <5 L/min. Parameters should be assessed and set by monitoring indicators such as end-tidal CO2 partial pressure (5–7).	5b	B
	29	At the end of surgery, CO2 supply should be stopped first. The surgeon should fully evacuate residual intra-abdominal gas before removing trocar puncture devices; this is retained as a pragmatic contamination-control precaution, whereas its ability to prevent port-site tumor-cell dissemination (the “chimney effect”) remains unproven (5, 7).	5b	B
	30	Heated and humidified CO2 insufflation may be considered to reduce intraoperative heat loss; evidence that warming CO2 reduces aerosolized tumor-cell dissemination is lacking, and the oncologic rationale stated in the previous version has been removed (12, 13).	5b	B

Evidence levels (2014 JBI Levels of Evidence): 1a = systematic review/meta-analysis of RCTs; 1b = individual RCT; 5b = expert consensus (evidence-based guidelines without a reported evidence level, expert consensus statements, and narrative reviews). Recommendation strength (FAME framework): Grade A = strong recommendation (all four FAME criteria satisfied); Grade B = weak recommendation.

## 4 Discussion

### 4.1 Specificity of barrier and no-touch techniques in GI surgery

Barrier techniques in GI surgery differ substantially from those in clean general surgery. Because GI procedures involve exposure to digestive contents and intestinal flora such as E. coli, Enterococcus, and Bacteroides spp., most incisions are clean-contaminated or contaminated and carry a markedly higher SSI risk. Existing authoritative guidelines (WHO (8), CDC (9), ASCRS (4) address SSI prevention for general or specialty-specific surgery but—with the exception of the colorectal-specific ASCRS guideline—do not provide GI-specific barrier protocols, forcing nurses to consult multiple documents and contributing to fragmented and inconsistent practice. This summary therefore integrates the available evidence into a unified, SSI-focused framework covering the entire intraoperative process of GI surgery.

### 4.2 Evidence-based recommendations for the NTIT

Critical appraisal of the NTIT evidence reveals an important controversy. Although biologically plausible, the 6-year follow-up of the JCOG1006 phase III trial (853 colon cancer patients) found no significant differences in disease-free (70.3% vs. 69.4%) or overall survival between NTIT and conventional techniques (1, 7). In our interpretation, the enduring value of NTIT-derived operational practices—zoned “clean-contaminated” instrument management and glove and instrument changes after anastomosis—lies less in proven oncologic benefit than in their contribution to contamination control and SSI prevention, which justifies their retention in this SSI-focused framework; either technique may therefore be selected according to surgeon experience. We emphasize, however, that this retention should not be misread as evidence of superiority: NTIT-derived statements in Table 6 (Domain 1) were therefore re-graded to reflect their actual evidence base—expert consensus (level 5b)—and are assigned weak (Grade B) recommendations, to be implemented as contamination-control practices rather than as oncologically beneficial techniques. In minimally invasive surgery, retrieval bag use is strongly recommended because specimen extraction through small incisions may contaminate the wound (23), whereas evidence on pneumoperitoneum-related dissemination and smoke filtration remains inconclusive and constitutes an important evidence gap.

### 4.3 Important value of wound protectors in SSI prevention in GI surgery

The strongest evidence identified in this best evidence summary concerns wound protectors. A 2025 meta-analysis of 19 RCTs (4,779 abdominal surgery patients) showed that wound protectors significantly reduce SSI (RR = 0.63, *P* = 0.0001), with a larger effect in the GI surgery subgroup (RR = 0.49, *P* < 0.00001; moderate-certainty evidence) (17), and a 2024 multicenter RCT confirmed an approximately 47% SSI risk reduction with double-ring plastic protectors vs. traditional gauze in open GI surgery (19); the ASCRS guideline recommends wound protectors in colorectal surgery4, whereas other included guidelines do not issue a specific recommendation for or against their use. We nevertheless note that most included trials enrolled open rather than laparoscopic procedures, so generalization to minimally invasive surgery remains uncertain. Cost and reimbursement barriers may also limit routine use in primary hospitals in China. We therefore propose tiered adoption—routine use of double-ring protectors in tertiary hospitals, and prioritized use of single-ring protectors for high-risk procedures (e.g., low anterior resection and emergency surgery for intestinal obstruction) in secondary hospitals as a transitional strategy—consistent with the feasibility dimension of the FAME framework. Only products certified under national medical device registration standards should be selected.

### 4.4 Application value of bundle measures in GI surgery SSI prevention

Perioperative care bundles are associated with a reduced risk of SSI (15), and the Chinese ERAS guidelines (2021) (13) endorse integrating multiple interventions into standardized protocols. Based on the synthesized evidence, we recommend a GI-surgery SSI prevention bundle comprising mechanical bowel preparation plus oral antibiotics for colorectal surgery (4, 22), standardized temperature management, timely preoperative antibiotic administration, intraoperative aseptic barrier and wound protection, 48-hour postoperative sterile dressing management, hand hygiene, and daily SSI risk assessment (4). Because the certainty of evidence differs across components, we further propose a “core-flexible” two-tier structure when designing such bundles: high-certainty measures (wound protectors, mechanical bowel preparation plus oral antibiotics, and temperature management) should constitute the core, whereas lower-certainty or uncertain-benefit measures (e.g., routine peritoneal irrigation, pneumoperitoneum smoke management, and NTIT-derived operational details) may be applied flexibly according to clinical conditions, so as to prioritize high-level evidence and avoid overly complex and unsustainable protocols.

### 4.5 Evidence translation and practice: challenges and recommendations

This best evidence summary has several limitations. Only Chinese- and English-language publications were searched, and gray literature may have been missed; only three RCTs were eligible, no eligible cohort studies were identified, and most NTIT-derived contamination-control practices are supported only by expert-consensus (level 5b) evidence, so the corresponding recommendations are necessarily weak (Grade B); blinding was incompletely reported in some primary studies. Important evidence gaps remain: guideline recommendations for laparoscopic and robotic barrier techniques are relatively general; high-level evidence on pneumoperitoneum management and trocar-site protection is lacking; and NTIT evidence derives predominantly from colon cancer, with limited data for gastric and rectal subgroups.

Future research should prioritize GI-specific barrier protocols for minimally invasive and robotic surgery (including retrieval bag use, trocar-site isolation, and pneumoperitoneum smoke evacuation); multicenter implementation-science studies evaluating barriers, facilitators, and cost-effectiveness of tiered, FAME-guided bundle strategies across hospital levels; and the development of tiered training curricula and competency-based assessment indicators for operating room nurses, supported by a whole-process quality monitoring system covering preoperative preparation, intraoperative cooperation, and postoperative handover.

## 5 Conclusions

This best evidence summary synthesized 18 high-quality publications into 30 best-evidence statements across six domains of intraoperative barrier and no-touch practice for SSI prevention in GI surgery. The strongest evidence supports routine use of wound protectors in open GI surgery, mechanical bowel preparation combined with oral antibiotics in colorectal surgery, maintenance of intraoperative normothermia; these measures should form the core of GI-surgery SSI-prevention bundles. Most NTIT-derived contamination-control practices are supported mainly by expert-consensus evidence and are conditionally recommended (Grade B) pending higher-quality studies; they should be implemented as contamination-control measures rather than as oncologically beneficial techniques. In contrast, evidence remains limited or conflicting for the oncologic benefit of the NTIT, pneumoperitoneum and smoke management, optimal irrigation regimens, and barrier practices specific to laparoscopic and robotic surgery; recommendations in these areas should be applied flexibly. Clinical translation should follow the FAME framework within a “core-flexible” two-tier structure adapted to hospital resources and individual patient conditions. Priorities for future research include GI-specific barrier protocols for minimally invasive and robotic surgery, validation in gastric and rectal cancer subgroups, and multicenter implementation-science studies of barriers, facilitators, and cost-effectiveness.

## Data Availability

The original contributions presented in the study are included in the article/Supplementary Material, further inquiries can be directed to the corresponding author.
